# The Arrhythmogenic Calmodulin p.Phe142Leu Mutation Impairs C-domain Ca^2+^ Binding but Not Calmodulin-dependent Inhibition of the Cardiac Ryanodine Receptor*

**DOI:** 10.1074/jbc.M116.766253

**Published:** 2016-12-07

**Authors:** Mads Toft Søndergaard, Yingjie Liu, Kamilla Taunsig Larsen, Alma Nani, Xixi Tian, Christian Holt, Ruiwu Wang, Reinhard Wimmer, Filip Van Petegem, Michael Fill, S. R. Wayne Chen, Michael Toft Overgaard

**Affiliations:** From the ‡ Department of Chemistry and Bioscience, Aalborg University, 9220 Aalborg, Denmark,; the § Libin Cardiovascular Institute of Alberta, the Department of Physiology and Pharmacology and the Department of Biochemistry and Molecular Biology, University of Calgary, Calgary, Alberta T2N 1N4, Canada,; the ‖ Department of Biochemistry and Molecular Biology, University of British Columbia, Vancouver, British Columbia V6T 1Z3, Canada, and; the ¶ Department of Molecular Biophysics and Physiology, Rush University Medical Center, Chicago, Illinois 60612

**Keywords:** calcium intracellular release, calcium-binding protein, calmodulin (CaM), protein-protein interaction, ryanodine receptor, CaM-F142L, arrhythmia, cardiac ryanodine receptor

## Abstract

A number of point mutations in the intracellular Ca^2+^-sensing protein calmodulin (CaM) are arrhythmogenic, yet their underlying mechanisms are not clear. These mutations generally decrease Ca^2+^ binding to CaM and impair inhibition of CaM-regulated Ca^2+^ channels like the cardiac Ca^2+^ release channel (ryanodine receptor, RyR2), and it appears that attenuated CaM Ca^2+^ binding correlates with impaired CaM-dependent RyR2 inhibition. Here, we investigated the RyR2 inhibitory action of the CaM p.Phe142Leu mutation (F142L; numbered including the start-Met), which markedly reduces CaM Ca^2+^ binding. Surprisingly, CaM-F142L had little to no aberrant effect on RyR2-mediated store overload-induced Ca^2+^ release in HEK293 cells compared with CaM-WT. Furthermore, CaM-F142L enhanced CaM-dependent RyR2 inhibition at the single channel level compared with CaM-WT. This is in stark contrast to the actions of arrhythmogenic CaM mutations N54I, D96V, N98S, and D130G, which all diminish CaM-dependent RyR2 inhibition. Thermodynamic analysis showed that apoCaM-F142L converts an endothermal interaction between CaM and the CaM-binding domain (CaMBD) of RyR2 into an exothermal one. Moreover, NMR spectra revealed that the CaM-F142L-CaMBD interaction is structurally different from that of CaM-WT at low Ca^2+^. These data indicate a distinct interaction between CaM-F142L and the RyR2 CaMBD, which may explain the stronger CaM-dependent RyR2 inhibition by CaM-F142L, despite its reduced Ca^2+^ binding. Collectively, these results add to our understanding of CaM-dependent regulation of RyR2 as well as the mechanistic effects of arrhythmogenic CaM mutations. The unique properties of the CaM-F142L mutation may provide novel clues on how to suppress excessive RyR2 Ca^2+^ release by manipulating the CaM-RyR2 interaction.

## Introduction

Point mutations in one of the three extremely conserved calmodulin (CaM)^2^-encoding genes, *CALM1–3*, result in life-threatening ventricular arrhythmias likely due to altered CaM-regulation of the ion channels that govern cardiac excitation-contraction (1–8). The CaM-N54I and -N98S mutations (numbering includes start-Met) were identified in individuals with catecholaminergic polymorphic ventricular tachycardia (CPVT). The CaM-D96V, -D130G, and -F142L mutations were found in individuals with long QT syndrome (LQTS) (1, 2). Interestingly, the CaM-N98S mutant also apparently causes LQTS or a mixed phenotype, depending on the genetic background (4). Not only do these various mutations impose different cardiac arrhythmias, but it appears that their disease mechanisms differ at the molecular level for each CaM target, even within the same arrhythmia type (6–11). One such CaM target is the cardiac Ca^2+^ release channel/ryanodine receptor (RyR2). RyR2 mediates Ca^2^ release from the sarcoplasmic reticulum (SR) in cardiomyocytes (12, 13). The RyR2 protein forms homotetrameric channels in the SR membrane with a large cytosolic domain that interacts with numerous proteins and ligands, which regulate RyR2 Ca^2+^ release (12, 13). During cardiac excitation-contraction coupling, RyR2 channels are activated by Ca^2+^ entry into the cytosol through sarcolemmal voltage-gated Ca^2+^ channels (Ca_V_1.2). This Ca^2+^ entry triggers RyR2-mediated SR Ca^2+^ release via a process called Ca^2+^-induced Ca^2+^ release, which results in the rise in cytosolic free Ca^2+^ ([Ca^2+^]_cyt_) and thereby drives contraction (14). RyR2 channels are sensitive to both [Ca^2+^]_cyt_ and the SR luminal free Ca^2+^ ([Ca^2+^]_SR_) as well as a plethora of regulatory signals (13). One of these regulatory signals is CaM binding to RyR2, which generally inhibits Ca^2+^ release both at the diastolic and systolic [Ca^2+^]_cyt_.

**FIGURE 1.**
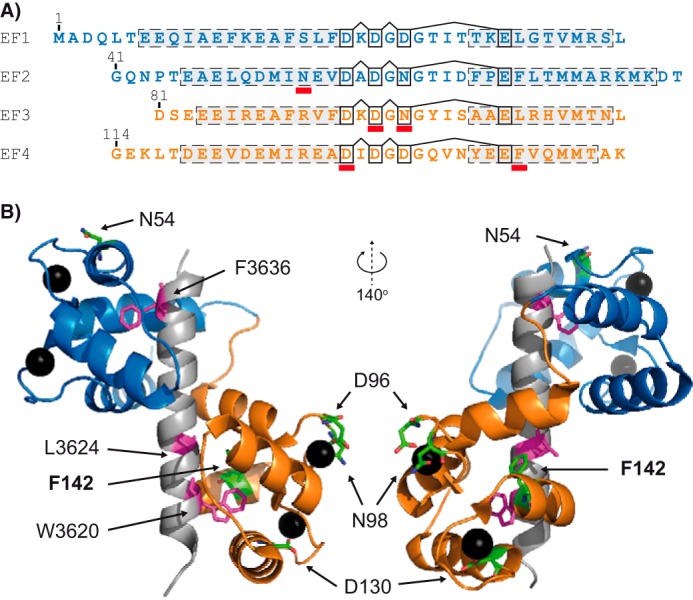
**Overview of CaM and selected CaM mutations.** *A*, the primary structure of CaM (N-domain, Met-1–Thr-80; C-domain, Asp-81–Lys-149) with EF-hands 1–4 aligned. *Dashed boxes* indicate α-helices and *connected black boxes* the Ca^2+^ coordinating residues. *Red bars* highlight individual sites for arrhythmogenic mutations (N54I, D96V, N98S, D130G, and F142L). *B*, representative tertiary structure of CaM. Ca^2+^-saturated CaM binding to a 27-residue peptide corresponding to part of the CaMBD in RyR1 (Lys-3614–Pro-3640) (Protein Data Bank code 2BCX) is shown. Protein and peptide secondary structures are represented schematically. The N-domain of CaM is indicated in *blue*, the C-domain in *orange*, and the peptide in *gray*. Sites of arrhythmogenic CaM mutations are highlighted as *green stick* representations (non-mutated residues) and Ca^2+^ ions as *black spheres*. RyR1 CaMBD residues (Trp-3620, Leu-3624, and Phe-3636) corresponding to RyR2 CaMBD residues Trp-3587, Leu-3591, and Phe-3603 are highlighted as *magenta stick* representations.

CaM is a ubiquitously expressed sensor of cytosolic Ca^2+^ signals that has two Ca^2+^-binding domains (N- and C-domain), each containing two EF-hand motifs. These two domains are separated by a flexible linker, and thus one CaM protein binds up to four Ca^2+^ (Fig. 1). The two domains of CaM display distinct affinities and kinetics for binding to Ca^2+^. This allows the CaM domains to have both independent and correlated interactions with different CaM targets (3, 15–17). Moreover, the Ca^2+^ binding attributes of either domain are affected by Ca^2+^ binding to the other domain as well as by the binding of CaM to protein targets (8, 17–20). In addition, protein complexes regulated by CaM generally contain more than one region for their interaction with the two CaM domains (3, 21, 22). For example, the binding of the CaM C-domain to the RyR2 CaM-binding domain (CaMBD) (Arg-3581–Pro-3607, human RyR2) is a prerequisite for CaM-dependent RyR2 inhibition. The RyR2 CaMBD also interacts with the CaM N-domain (Fig. 1), although less is known about this interaction (3, 8, 23–26). The pivotal role of this CaMBD in CaM-dependent RyR2 inhibition has been demonstrated unequivocally, but some studies suggest that other putative CaMBD in RyR2 may be involved as well (8, 26–29).

Recently, we showed that both the CPVT-causing CaM-N54I and the CPVT- and LQTS-causing CaM-N98S, as well as the LQTS-causing CaM-D96V and -D130G mutations markedly reduce inhibition of RyR2 Ca^2+^ release during store overload-induced Ca^2+^ release (SOICR) (8). The CaM-D96V, -N98S, and -D130G mutations directly affect Ca^2+^-coordinating residues in the C-domain. Thus, the diminished ability of these mutations to regulate RyR2 function is potentially explained by the reduced C-domain Ca^2+^ binding. Unlike the CaM-D96V, -N98S, and -D130G mutations, CaM-F142L does not affect a Ca^2+^-coordinating residue but still reduces CaM C-domain Ca^2+^ binding (2). In the X-ray structure of CaM complexed with the RyR1 CaMBD, the Phe-142 residue directly contributes to the CaM-CaMBD binding interface in contrast to the CaM Asn-54, Asn-98, Asp-96, and Asp-130 residues. We used a combination of functional, biophysical, and structural assays to investigate in detail the action of the LQTS-causing CaM-F142L mutation on RyR2 regulation. Unexpectedly, we found that the F142L mutation caused only a minor reduction in RyR2 inhibition by CaM (compared with CaM-WT), despite the markedly reduced CaM-F142L C-domain Ca^2+^ binding. Even more surprisingly, the F142L mutation enhanced the inhibitory action of CaM in RyR2 single channel experiments (*i.e.* displayed a CaM gain-of-function (GoF) effect). These actions are unique to the CaM-F142L mutation as compared with the CaM-N54I, -D96V, -N98S, and -D130G mutations. Discovery of this unique GoF property, conferred by the CaM-F142L mutation, may potentially serve as a molecular guide for how to manipulate CaM-dependent RyR2 inhibition as a therapeutic strategy for treating arrhythmias and/or heart failure.

## Results

### 

#### 

##### The CaM-F142L Mutation Slightly Decreases the Termination Threshold for Store Overload-induced Ca^2+^ Release

**FIGURE 2.**
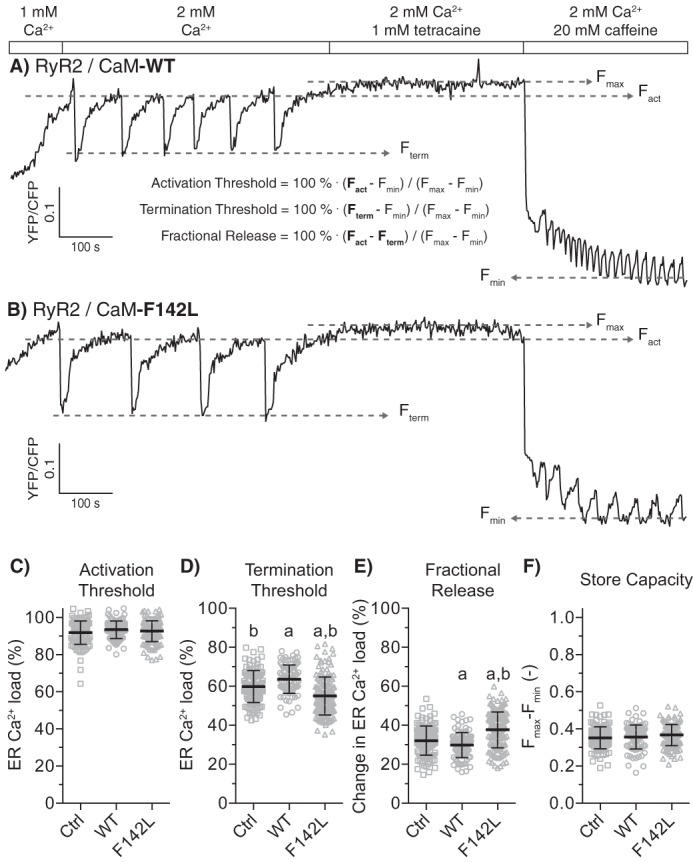
**CaM F142L minutely affects regulation of RyR2 Ca^2+^ release during SOICR.** *A* and *B*, example traces for the D1ER signal during SOICR from RyR2 expressing HEK293 cells co-transfected with CaM-WT (*A*) or CaM-F142L (*B*). The FRET signal from the ER luminal D1ER [Ca^2+^]_free_ indicator oscillated as Ca^2+^ was released through RyR2. The *rectangular bars* are labeled to indicate the concentrations of Ca^2+^, tetracaine (RyR2 channel inhibitor), and caffeine (activator) in the perfusion solution. The increase in the ER Ca^2+^ concentration elicited RyR2 SOICR oscillations; the tetracaine blocked Ca^2+^ release, filling ER to the maximum [Ca^2+^]_free_; and finally caffeine depleted ER Ca^2+^ to the minimum [Ca^2+^]_free_ (*F*_max_ and *F*_min_). *C–F*, the activation (*C*) and termination (*D*) thresholds and the fractional Ca^2+^ release (*E*) relative to *F*_max_ and *F*_min_; their difference was the fractional ER Ca^2+^ release (*F*). The expression of CaM-WT or -F142L affected the measured termination thresholds compared with endogenous CaM (Ctrl). *Error bars* show S.D., and values were calculated from 90–140 single cell traces. The *a* and *b* indicate values significantly different from those for the Ctrl or CaM-WT (one-way ANOVA, *p* < 0.05), respectively.

To test whether the CaM-F142L mutation affects the regulation of RyR2 during SOICR, we transfected RyR2-expressing HEK293 cells with CaM-WT or -F142L and then monitored the endoplasmic reticulum (ER) Ca^2+^ concentration using the D1ER Ca^2+^ probe (27). Perfusion of the transfected cells with increasing extracellular Ca^2+^ concentrations induced SOICR in the form of spontaneous ER Ca^2+^ oscillations (Fig. 2) (27, 30). The oscillating D1ER signal was then used to determine the ER Ca^2+^ level at which SOICR occurred (activation threshold) and the ER Ca^2+^ depletion at which SOICR ended (termination threshold; Fig. 2*A* and “Experimental Procedures”). The difference between the activation and termination thresholds was specified as the fractional ER Ca^2+^ release. Fig. 2, *B* and *D*, shows that expression of the CaM-F142L mutant decreased the termination threshold by 5% compared with the control with only endogenous CaM-WT (F142L 55% *versus* control 60%, *p* < 0.001). This in turn increased the fractional ER Ca^2+^ release during Ca^2+^ release oscillations by 6% (Fig. 2*E*, F142L 38% *versus* control 32%, *p* < 0.001). Note that the percentages listed here refer to the unit for ER Ca^2+^ load and not the relative effects of the CaM variants. On the other hand, expression of CaM-WT increased the termination threshold by 4% (WT 64% *versus* control 60%, *p* < 0.01) and minutely reduced the fractional ER Ca^2+^ release (WT 30% *versus* control 32%, p ≈ 0.1), although the latter was not statistically significant (Fig. 2, *A* and *E*). Neither CaM-WT nor CaM-F142L expression affected the activation threshold (Fig. 2, *A–C*). The control and CaM-WT results here are highly consistent with those reported previously (27). Taken together, CaM-F142L slightly reduced the SOICR termination threshold or, in other words, had a slightly less inhibitory action on RyR2-mediated Ca^2+^ release compared with CaM-WT. For comparison, we previously reported equivalent experiments showing that CPVT-causing (N54I), CPVT- and LQTS-causing (N98S), and LQTS-causing CaM mutations (D96V and D130G) all dramatically alter the Ca^2+^ release termination threshold (8). Specifically, the CaM-N54I, -D96V, -N98S, and -D130G mutations reduced the termination threshold by 12, 17, 18, and 16%, respectively. These variants also minimally, but significantly, reduced the activation threshold (∼5%) (8). Thus, the action of the CaM-F142L mutant on RyR2 function appeared very different from that of CaM-N54I, -D96V, -N98S, and -D130G.

##### The CaM-F142L Mutation Enhances Inhibition of Single RyR2 Channels

**FIGURE 3.**
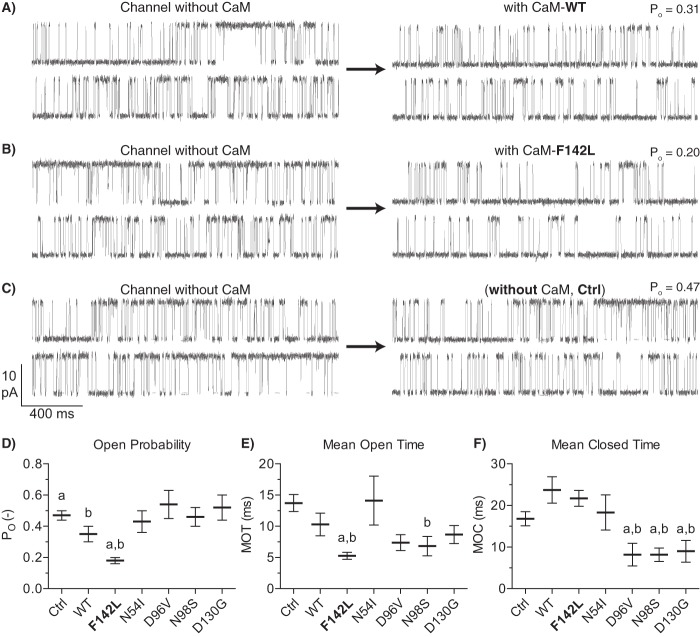
**Effect of CaM-WT and mutants on single RyR2 channel properties.** *A–C*, example of single RyR2 channel current traces without CaM (*left traces*) and after the addition of 1 μm CaM. *D–F*, RyR2 channel *P_O_*, MOT, and MCT after the addition of CaM mutants (Ctrl with no added CaM). Measurements were done at +40 mV with 1 mm free Ca^2+^ at the luminal face and 10 μm free Ca^2+^, 1 mm free Mg^2+^, and 1 mm ATP at the cytosolic face. The *a* indicates values significantly different from CaM-WT, and *b* indicates values significantly different from Ctrl (*t* test, *p* < 0.05). *Error bars* show S.E. from 6–8 channels for each CaM variant and 32 channels before or without CaM addition (Ctrl).

Next, we tested the action of the CaM mutations on single RyR2 channels incorporated into lipid bilayers. Luminal [Ca^2+^]_free_ was kept at 1 mm, and the cytosolic [Ca^2+^]_free_ was set at 10 μm. The cytosolic solution also contained 1 mm [Mg^2+^]_free_ and 5 mm ATP to approximate the levels present in cells. The 10 μm cytosolic [Ca^2+^]_free_ approximates the level that RyR2 encounter during systole (see “Experimental Procedures”). Single RyR2 channel function was measured before and after the addition of 1 μm CaM-WT, -F142L, -N54I, -D96V, -N98S, or -D130G to the cytosolic solution (Fig. 3). Control (Ctrl) recordings in the absence of CaM were also included. The addition of CaM-WT significantly lowered the RyR2 open probability (*P_O_*) from 0.47 to 0.35 compared with control, consistent with CaM-WT inhibition of RyR2-mediated Ca^2+^ release (Fig. 3*D*). CaM-WT reduced the mean open time (MOT) and increased the mean closed time (MCT), although these changes individually were not statistically significant (*p* = 0.18 and 0.05 against Ctrl) (Fig. 3, *E* and *F*). In contrast, the C-domain mutations, CaM-D96V, -N98S, and -D130G, did not lower RyR2 *P_O_* compared with the control (*P_O_* 0.54, 0.46, and 0.52, respectively). The CaM-D96V, -N98S, and -D130G mutations all significantly decreased the RyR2 MCT compared with both CaM-WT and the control. The CaM-N98S mutation significantly decreased MOT compared with the control. These results suggest that: 1) these CaM mutations do interact with RyR2 as also shown in previous studies (7, 8, 10); and 2) CaM mutations D96V, N98S, and D130G, in contrast to CaM-WT, do not appear to inhibit RyR2 function (but this is due to almost equal decreases in both MOT and MCT compared with no CaM present). In contrast, the CaM N-domain N54I mutation did not significantly affect RyR2 function (*P_O_*, MCT or MOT) under these experimental conditions, suggesting that aberrant RyR2 regulation by CaM-N54I is mechanistically distinct from that caused by the CaM C-domain mutations (8, 17).

Strikingly, the C-domain CaM-F142L mutation had an opposite action on single RyR2 channel function. CaM-F142L significantly lowered the RyR2 *P_O_*, even more so than CaM-WT (*P_O_* 0.18 *versus* 0.35) (Fig. 3*D*). The CaM-F142L mutation caused a significant MOT decrease compared with both CaM-WT and the control, yet appeared to have approximately the same effect on MCT as CaM-WT (22 *versus* 24 ms). Hence, the CaM-F142L mutation resulted in a stronger RyR2 inhibitory action (compared with that of CaM-WT). In other words, the CaM F142L mutation generates a GoF defect with regard to RyR2 inhibition, which is in stark contrast to CaM-D96V, -N98S, and -D130G, none of which inhibited RyR2 function. Hence, CaM-WT, CaM-F142L, and the other C-domain mutations (D96V, N98S, and D130G), as well as the CaM N-domain N54I mutation tested here, have distinctive actions on RyR2 modulation.

##### The CaM-F142L Mutation Reduces Ca^2+^ Binding Affinity in the Presence of the RyR2 CaMBD Peptide

**FIGURE 4.**
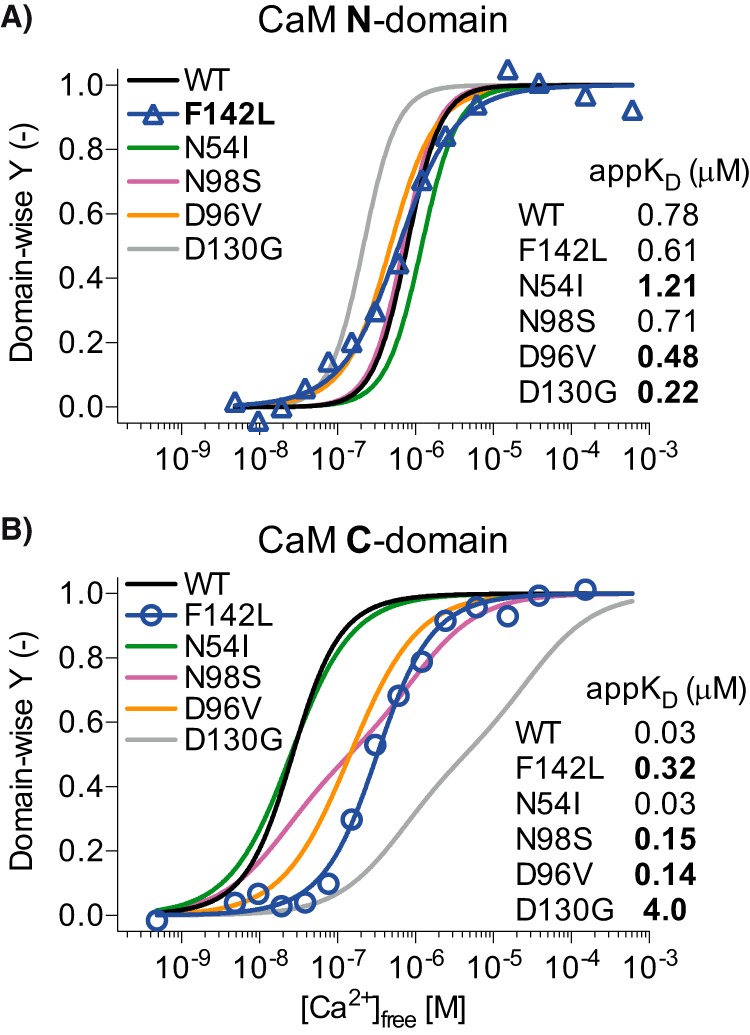
**Domain-specific titration curves for Ca^2+^ binding to CaM in the presence of RyR2(R3581-L3611) peptide.** CaM-F142L/RyR2(R3581-L3611) Ca^2+^ titration points for CaM N-domain (*A*, *triangles*) and C-domain (*B*, *circles*) were fitted with a two-site Adair model (*solid blue lines*), and normalized *Y* (see “Experimental Procedures”) was plotted as a function of [Ca^2+^]_free_. The *solid lines* in either *panel* show the fit to the equivalent titrations of CaM-WT (*black*), -N54I (*green*), -N98S (*magenta*), -D96V (*tangerine*), and -D130G (*gray*) with data from Søndergaard *et al*. (8). *Bold*, *highlighted* app*K_D_* values were significantly different from those for CaM-WT/RyR2(R3581-L3611) (one-way ANOVA *p* < 0.05).

The ability of CaM to inhibit RyR2 Ca^2+^ release depends both on Ca^2+^ binding to CaM and CaM binding to the RyR2 CaMBD (2, 29, 31). The CaM-F142L mutation clearly impairs Ca^2+^ binding to the CaM C-domain in the absence of a CaM binding target (2, 29, 31). Given the limited action of the CaM-F142L mutant on SOICR in HEK293 cells and its GoF action at the single RyR2 channel level, we assessed whether the CaM-F142L mutation differentially affects CaM Ca^2+^ affinity when the CaM is bound to the RyR2 CaMBD. The binding of Ca^2+^ to CaM in the presence of the CaMBD (*i.e.* the RyR2(R3581-L3611) peptide) was determined using a Ca^2+^ titration while monitoring the intrinsic protein fluorescence specific for each CaM domain (Phe for the N-domain and Tyr for the C-domain) (Fig. 4). Fitting the apparent dissociation constant (app*K_D_*) for either CaM domain using a two-site Adair model revealed that the CaM-F142L mutation lowered the affinity of the C-domain for binding Ca^2+^ in the presence of the CaMBD more than 10-fold compared with CaM-WT (app*K_D_* 0.32 *versus* 0.03 μm). The CaM-F142L mutation did not significantly affect N-domain Ca^2+^ binding, albeit there was a tendency toward increased affinity (app*K_D_* 0.61 *versus* 0.78 μm). Moreover, the CaM-F142L C-domain Ca^2+^ binding affinity in the presence of the RyR2 CaMBD appeared ∼2-fold greater than that observed for the CaM-N98S and -D96V mutations (app*K_D_* 0.15 and 0.14 μm, respectively). Note that in the absence of a CaM target, the CaM-F142L mutant C-domain Ca^2+^ binding has an affinity intermediate of these other mutations (app*K_D_* 10, 15, and 31 μm for CaM-N98S, -F142L, and -D96V, respectively) (2, 8). Thus, the CaM-F142L mutation causes a loss of function (LoF) in terms of Ca^2+^ binding to the CaM C-domain when complexed with its RyR2 target, and this LoF is at least as severe as for CaM-D96V or -N98S.

##### Thermodynamically Distinct Interactions of CaM-F142L and CaM-WT with the RyR2 CaMBD

**FIGURE 5.**
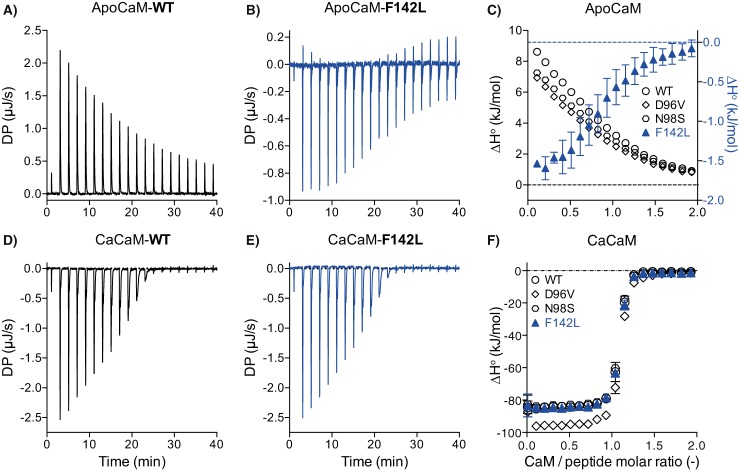
**ITC example data for the titration of the RyR2(R3581-P3607) peptide with CaM.** Example ITC thermograms for apoCaM-WT and -F142L (*A* and *B*) and for CaCaM conditions (*D* and *E*). *C* and *F*, averaged integrated change in enthalpy per injection (Δ*H*°) comparing CaM-WT, -D96V, -N98S, and -F142L under apo (*C*) or CaCaM conditions (*F*). Without Ca^2+^ present, the binding of apoCaM-WT to the peptide is an endothermic reaction (positive Δ*H*°), whereas for the apoCaM-F142L the interaction is exothermic (*A* and *B*). With saturating Ca^2+^ present, both CaCaM-WT and -F142L display exothermic binding reactions (*D* and *E*). Thermograms for CaM-D96V and -N98S were not visibly different from those for the CaM-WT and therefore are not shown. *DP*, change in ITC instrument heat effect.

**FIGURE 6.**
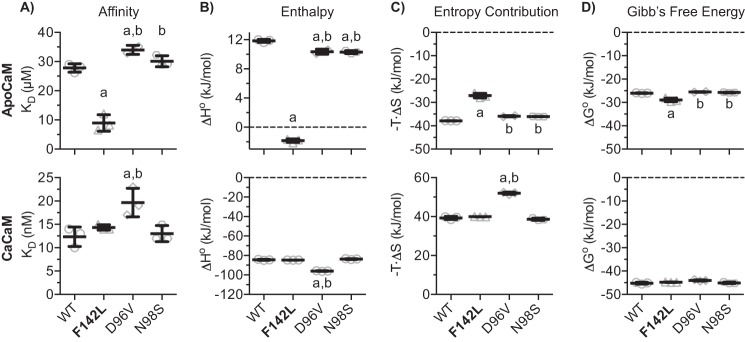
**Fitted thermodynamic parameters for the binding of CaM variants to the RyR2(R3581-P3607) peptide as calculated from ITC.** *Panels* in the *upper row* summarize values for the apoCaM condition, and those in the *lower row* summarize values for CaCaM. *A*, the affinity of CaM for binding to the peptide expressed as the dissociation constant (*K_D_*). *B* and *C*, enthalpy (Δ*H*°) and entropy contribution (−*T*·ΔS°), respectively, for the binding interaction between CaM and the peptide. *D*, the change in Gibb's free energy (Δ*G*°) as conferred by the CaM mutations. *Error bars* show S.D. The *a* and *b* indicate values significantly different from those for CaM-WT or CaM-F142L, respectively (one-way ANOVA, *p* < 0.05).

**FIGURE 7.**
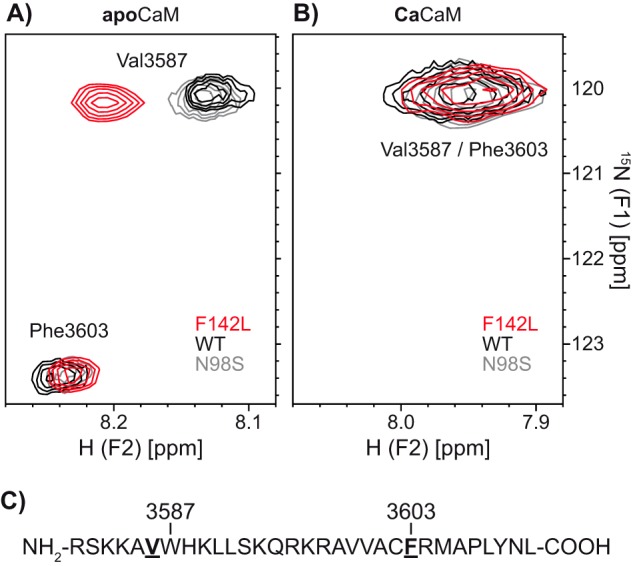
**2D [^15^N,^1^H]HSQC NMR spectra of CaM in complex with the RyR2(R3581-L3611) peptide.** ^15^N-hydrogen HSQC NMR spectra of the RyR2(R3581-L3611) peptide with ^15^N-labeled Val-3586 and Phe-3603 in complex with CaM-WT, -N98S, or -F142L. The *labeled residues* correspond to Phe-3636 and the Val adjacent to Trp-3620 highlighted in Fig. 1. Spectra were recorded without any Ca^2+^ (*A*) and with saturating Ca^2+^ (*B*). The *peaks* are color-coded according to the CaM variant in the protein-peptide complex. *C*, amino acid sequence of the RyR2(R3581-L3611) peptide with ^15^N-labeled residues highlighted in *bold* and *underlined*. The CaM C-domain binds around Trp-3587 and the N-domain around Phe-3603.

To further probe the interaction between the CaM-F142L mutant and the RyR2 CaMBD, we followed the titration of CaMBD (*i.e.* the RyR2(R3581-P3607) peptide) with CaM variants under Ca^2+^-free (apoCaM) and saturating Ca^2+^ conditions (CaCaM) using ITC (Fig. 5). This measurement compares the differences in the CaMBD interaction aside from those caused by differences in CaM Ca^2+^ binding affinity. The binding of apo- or CaCaM-WT to the CaMBD peptide represent two thermodynamically distinct reactions. The apoCaM-WT/CaMBD interaction is comparatively low affinity (μm *K_D_*) and is entropy-driven (Δ*H*° > 0, −*T*·ΔS° < 0). The CaCaM-WT/CaMBD interaction is high affinity (nm *K_D_*) and is enthalpy-driven (Δ*H*° ≪ 0, −*T*·ΔS° > 0) (Figs. 5–7) (26).

The ITC measurements showed a fundamental difference in the thermodynamics of apoCaM-F142L binding to the CaMBD compared with the apoCaM-WT (Fig. 5, *A–C*). The binding reaction between apoCaM-F142L and the CaMBD peptide was exothermic (Δ*H*° < 0) in marked contrast to the endothermic (Δ*H*° > 0) interaction between apoCaM-WT and the CaMBD. Moreover, titration curve analysis showed that apoCaM-F142L binding affinity for the CaMBD was ∼3-fold greater compared with that of apoCaM-WT (*K_D_* 9 *versus* 28 μm) (Fig. 6*A*). ApoCaM-F142L binding also displayed a small negative Δ*H*° (−1.8 kJ/mol)) compared with the larger, positive Δ*H*° (11.8 kJ/mol) for apoCaM-WT binding (Fig. 6*B*). A comparison of the fitted Δ*H*° values to the −*T*·ΔS° values (−38 and −27 kJ/mol for apoCaM-WT and -F142L) indicated that the binding of both apoCaM-WT and -F142L to the CaMBD peptide remained governed by entropy (−*T*·ΔS°) relative to enthalpy (Δ*H*°) (Fig. 6*C*). The change in Δ*H*° conferred by the CaM-F142L mutation translated into a significant 11% decrease (−29 *versus* −26 kJ/mol) in Δ*G*° for the apoCaM-F142L interaction with the CaMDB peptide (Fig. 6*D*). Generally, an enthalpy-driven reaction is indicative of specific molecular bonding, whereas an entropy-driven reaction indicates hydrophobic interactions and solvent effects (32). Thus, these ITC results indicated that the CaM-F142L mutation transformed the apoCaM interaction from mainly entropy-driven to one more dominated by molecular bonds. Under saturating Ca^2+^ conditions, the interaction between CaCaM-F142L and the CaMBD peptide was indistinguishable from that for the CaCaM-WT (Figs. 5, *C* and *D*, and 6). Interestingly, the increase in affinity comparing Ca^2+^-free to saturating Ca^2+^ conditions was still 3 orders of magnitude (*K_D_* 8 μm *versus* 14 nm) for CaM-F142L, attributable to an increased Δ*H*° contribution under the CaCaM condition. Thus, the thermodynamic difference between apo and CaCaM binding to the CaMBD peptide remained similar for CaM-WT and -F142L.

The interactions of CaM-D96V and -N98S with the RyR2 CaMBD peptide were also investigated using ITC, and for both Ca^2+^ conditions neither of the thermograms (not shown) was visibly different from those for the CaM-WT. However, detailed titration curve analysis indicated a slightly decreased affinity of CaM-D96V for CaMBD binding with and without Ca^2+^ present (Figs. 5, *C* and *F*, and 6*A*). Small changes to Δ*H*° were also detected for apoCaM-D96V and -N98S and for CaCaM-D96V and -N98S (Fig. 6*B*). Interestingly, the minute effects observed for CaM-D96V and -N98S were all significantly distinct from those observed for CaM-F142L under both Ca^2+^ conditions. Thus, CaM-F142L generally showed CaMBD binding properties different from not only the CaM-WT but also from the other arrhythmogenic CaM mutations, D96V and N98S.

##### ApoCaM-WT and -F142L Binding to RyR2 CaMBD Peptide Are Structurally Distinct Interactions

The apoCaM C-domain binds to RyR2 CaMBD around Trp-3587 and Leu-3591. Upon CaM C-domain Ca^2+^ binding, this interaction shifts toward Trp-3587 (1, 8, 23, 25, 26, 33). The CaCaM N-domain binds to the RyR2 CaMBD peptide around Phe-3603, but it is unclear whether this interaction occurs for the apoCaM N-domain or is dependent on N-domain Ca^2+^ binding (3, 8, 27).

Motivated by the results from the ITC experiment, we used 2D NMR (^15^N-HSQC) to measure the effect of Ca^2+^ on the chemical shifts from a CaMBD peptide with ^15^N-labeled Val-3586 and Phe-3603 in a complex with CaM-WT, -N98S, or -F142L. The chemical shifts of these labeled residues depend on their immediate structural surroundings (*i.e.* the binding of the CaM C- and N-domain, respectively). The peaks corresponding to Val-3586 and Phe-3603 were easily discernable under apoCaM conditions (Fig. 7*A*). Unfortunately, their chemical shifts overlapped under saturating Ca^2+^ conditions (Fig. 7*B*). Nonetheless, a clear difference under apoCaM conditions was observed for CaM-CaMBD complexes containing CaM-F142L compared with CaM-WT or -N98S. ApoCaM-F142L displayed a higher chemical shift for the Val-3586 H^N^ but showed no differences for the Phe-3603 H^N^ or N. The latter observation may reflect that the N-domains of apoCaM-F142L and -WT do not bind to the CaMBD peptide. Under saturating Ca^2+^ conditions, no differences in the spectra were observed, albeit the addition of Ca^2+^ clearly affected the structural surroundings of the labeled residues (*i.e.* chemical shifts for both Val-3586 and Phe-3603 changed markedly). Opposite CaM-F142L, the spectra recorded using CaM-N98S were identical to those for CaM-WT, with and without Ca^2+^ (Fig. 7, *A* and *B*). Taken together, these results support the notion that the apoCaM-F142L C-domain binds to the RyR2 CaMBD peptide close to the Val-3586 residue (next to Trp-3587) in a unique conformation structurally distinct from that of the apoCaM-WT and -N98S. Further, Ca^2+^ binding to CaM-F142L changes this conformation into one that is indistinguishable from that of the CaCaM-WT and -N98S.

## Discussion

CaM is a constitutive Ca^2+^ sensor of the RyR2 macromolecular complex, where it inhibits RyR2 Ca^2+^ release in a [Ca^2+^]_cyt_ dependent, allosteric manner (3, 13, 34). This inhibition is critical for maintaining a low RyR2 activity at diastole (*i.e.* at low [Ca^2+^]_cyt_) and also for a sufficient termination of the Ca^2+^ release during cardiac excitation (*i.e.* as [Ca^2+^]_cyt_ increases) (8, 23, 27–29, 33, 35, 36). The details of how the two Ca^2+^-sensing domains of CaM interact with Ca^2+^ and RyR2 to facilitate this complex inhibition are not well understood (3, 13, 26, 34). However, the interactions between the RyR2 CaMBD, the CaM C-domain and Ca^2+^ are critical for the physiological regulation of RyR2 Ca^2+^ release (8, 23, 24, 27–29, 36). Binding of the apoCaM C-domain to the CaMBD inhibits RyR2 Ca^2+^ release, whereas binding of Ca^2+^ to the CaM C-domain further increases this CaM-dependent inhibition (8, 23, 27–29, 35, 36). Based on the biophysical results for the tripartite interaction (CaM, RyR2 CaMBD, and Ca^2+^) and the effects of arrhythmogenic CaM mutations, we proposed previously that the CaM C-domain at diastolic [Ca^2+^]_cyt_ binds to RyR2 CaMBD in a near Ca^2+^-saturated state (1, 8, 27). In this scheme, the reduced affinity for Ca^2+^ binding of the arrhythmogenic CaM C-domain mutations (D96V, N98S, and D130G) causes the CaM C-domain to be less Ca^2+^-saturated and thereby reduces the CaM-dependent inhibition of RyR2 (8).

**FIGURE 8.**
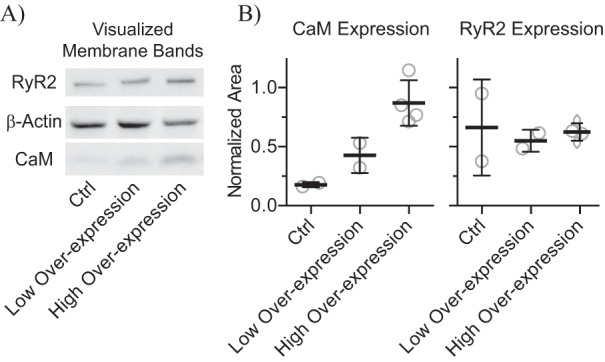
**Estimation of CaM and RyR2 expression levels in HEK293 cells.** *A*, example of Western-blotted protein bands visualized using chemiluminescence imaging. HEK293 cells were cultured with CaM-WT overexpression from a low or high expression plasmid and also without overexpression (Ctrl). *B*, the expression levels of CaM and RyR2 were normalized to that of β-actin. Averaged CaM expression levels were 0.18, 0.43, and 0.87 for endogenous CaM expression (Ctrl), and high and low plasmid expression, respectively. Opposite from CaM, the stable and inducible RyR2 expression levels appeared constant across all samples.

The LQTS-causing CaM-F142L mutation clearly reduces the C-domain Ca^2+^ binding affinity (app*K_D_* 15 μm), compared with CaM-WT (app*K_D_* 2.5 μm). This is similar to what was observed for the CaM-N98S and -D96V mutants (app*K_D_* 10 and 31 μm), albeit less than for CaM-D130G (app*K_D_* 84 μm) (2, 17). Here, we found that this reduced affinity for the CaM-F142L C-domain Ca^2+^ binding was retained in the presence of the RyR2 CaMBD peptide, even to the extent that CaM-F142L displayed a lower affinity than CaM-D96V (app*K_D_* 0.32 *versus* 0.14 μm) (Fig. 4). Thus, we expected that in HEK293 cells where [Ca^2+^]_cyt_ oscillates between ∼0.1 and 2 μm, CaM-F142L would abnormally regulate RyR2 function (compared with CaM-WT) to a similar extent as CaM-N98S and -D96V (Fig. 2) (37, 38). Curiously, the action of CaM-F142L on RyR2 function in the HEK293 SOICR assay was relatively benign and distinctly different from the actions of the CaM-N54I, -D96V, -N98S, and -D130G mutants (8). Moreover, these other CaM mutants were expressed using low expressing plasmids, resulting in a CaM ratio of ∼1.4 relative to endogenous CaM (8). The expression plasmid used in this study, and also in Tian *et al*. (27), results in a CaM ratio of ∼4 relative to endogenous CaM as judged from Western blotting analysis (Fig. 8). This further supports that the decrease in RyR2 inhibition caused by CaM-F142L was strikingly less than the decreases caused by CaM-N54I, -D96V, -N98S, and -D130G. Lastly, we observed no difference between CaM-F142L and -WT when the low expressing plasmid was used (data not shown). Consistent with these results, Hwang *et al.* (7) report similar spontaneous Ca^2+^ wave frequencies in permeabilized cardiomyocytes ([Ca^2+^]_free_ at 0.12 μm) with CaM-WT or CaM-F142L present. Also, Vassilakopoulou *et al.* (10) report that CaM-F142L inhibits binding of [^3^H]ryanodine to porcine cardiac SR vesicles to the same extent as the CaM-WT. Taken together, these results indicate that the F142L mutation causes little or no loss of CaM-dependent RyR2 inhibition, despite a pronounced LoF in terms of CaM C-domain Ca^2+^ binding. Even more strikingly though, we showed that at 10 μm cytosolic [Ca^2+^]_free_, CaM-F142L is a more potent inhibitor of single RyR2 channels than CaM-WT. Specifically, CaM-F142L promoted a much faster RyR2 closing (Fig. 3). In other words, CaM-F142L displayed a GoF action in that it increased the CaM-dependent RyR2 inhibition.

How can the F142L mutation confer both a LoF in terms of Ca^2+^ binding with seemingly little consequence to the RyR2 inhibitory action in cells and a robust RyR2 inhibitory action at the single channel level? Our ITC experiments hint at a potential explanation. ApoCaM-F142L showed increased affinity for binding the RyR2 CaMBD peptide and bound in a manner thermodynamically in between that of the apoCaM-WT and CaCaM-WT interactions (Figs. 5 and 6). The NMR HSQC spectra also support this view demonstrating that the apoCaM-F142L C-domain bound around Val-3586 in the CaMBD in a conformation that was structurally distinct from that of the apoCaM-WT. Based on our functional, biophysical, and structural results, we propose that the F142L mutation has two opposing actions on RyR2 regulation.

First, the F142L mutation enhances the CaM C-domain interactions with the RyR2 CaMBD, thus increasing RyR2 inhibition (GoF). Second, the CaM-F142L C-domain has impaired Ca^2+^ binding (LoF), thus decreasing RyR2 inhibition. Under the comparatively low [Ca^2+^]_cyt_ conditions in HEK293 cells or the permeabilized cardiomyocytes (7), the effect of reduced CaM-F142L C-domain Ca^2+^ binding would be partially offset by the enhanced CaM binding to the RyR2 CaMBD, explaining why little aberrant RyR2 regulation was observed. However, increasing Ca^2+^ saturates the CaM-F142L C-domain (app*K_D_*: 0.32 μm), in effect ablating any LoF action from the reduced C-domain Ca^2+^ affinity. Accordingly, at high Ca^2+^ concentrations only the GoF action would remain and may explain why CaM-F142L was a more potent inhibitor than the CaM-WT in our single RyR2 channel studies. The molecular basis for this GoF effect was not clear from these experiments, as no differences between CaCaM-WT and CaCaM-F142L binding to the RyR2 CaMBD peptide were detected. This implies perhaps that interactions between CaM and RyR2 not recapitulated in our biophysical experiments, *e.g.* RyR2 regions outside the CaMBD studied here, are responsible for the GoF action. Structurally delineating this GoF action may provide a molecular guide for how to manipulate the CaM-dependent RyR2 inhibition. Also, increasing this inhibition reduces SR Ca^2+^ release and/or leak and thus represents a therapeutic approach for treating heart failure and arrhythmia (28, 31, 39–41).

Aside from the novel insights into the effects of CaM-F142L on RyR2 regulation, our single RyR2 channel experiments support our previous finding that both CPVT- and LQTS-causing CaM mutations (N98S, D96V, and D130G) result in aberrant RyR2 regulation (8). Using different experimental conditions (sheep cardiomyocytes, 0.1 mm luminal [Ca^2+^]_free_, 2 mm ATP cytosolic, and no Mg^2+^), Hwang *et al.* (7) report that CaM-N54I and -N98S increased RyR2 *P_O_* at 0.1 and 1 μm cytosolic [Ca^2+^]_free_, whereas CaM-D96V did not. The apparent CaM-D96V discrepancy between our study and the Hwang *et al.* study (7) may be explained by differences in the experimental conditions. Our study shows that CaM mutations N54I, D96V, N98S, D130G, and F142L alter the RyR2 CaM regulation via distinct molecular mechanisms. The CaM-D96V, -N98S, and -D130G mutations diminish C-domain Ca^2+^ binding and thereby the inhibitory interaction of CaM with the RyR2 CaMBD. The N-domain CaM-N54I mutation likely affects CaM-RyR2 interactions that are outside the canonical CaMBD and/or increases Ca^2+^ binding kinetics (17). The CaM-F142L mutation diminishes C-domain Ca^2+^ binding but also enhances CaM interaction with the RyR2 CaMBD and possibly other regions (7, 8, 10, 17). Because LQTS-causing CaM-D96V, -N98S, and -D130G mutations diminish CaM-dependent RyR2 inhibition, increased RyR2 Ca^2+^ release may contribute to the LQTS phenotypes in individuals with these CaM mutations. Generally, it is thought that spontaneous diastolic RyR2 Ca^2+^ release causes CPVT, and augmented Ca^2+^ influx through Ca_V_1.2 channels causes LQTS (see Refs. 42 and 43 for details). Studies using recombinant Ca_V_1.2 and patch clamping show that Ca^2+^-dependent inactivation of Ca_V_1.2 is reduced when CaM-D96V, -N98S, -D130G and -F142L are present (compared with CaM-WT) (6, 9).

Thus, some mechanistic overlap between CPVT and LQTS caused by CaM mutations likely exist, and how arrhythmogenic CaM mutations manifest probably depends on their relative effects on RyR2, Ca_V_1.2, and other CaM-regulated targets. The best example of this is CaM-N98S, which affects both RyR2-mediated Ca^2+^ release and Ca_V_1.2 Ca^2+^-dependent inactivation, likely contributing to both CPVT and LQTS. Another scenario is represented by the strictly CPVT-causing CaM-N54I mutation, which does not affect Ca_V_1.2 Ca^2+^-dependent inactivation (1, 4, 6, 44). In addition, some CaM C-domain mutations (N98S, D132E, and Q136P) are reported to cause a LQTS phenotype with some features of CPVT overlapping (4, 44). β-Blockers are the common drug treatment for both CPVT and LQTS (42, 43). Given the differential influence of CaM mutants on Ca_V_1.2 and RyR2, drugs that preferentially alter RyR2 or Ca_V_1.2 function might provide better treatments for CaM-mediated arrhythmias (42, 43, 45). Novel drugs affecting RyR2 and Ca_V_1.2 are being sought (45–47), and as antiarrhythmic therapies advance, defining their differential action on Ca_V_1.2 and RyR2 function will almost certainly become increasingly relevant.

## Experimental Procedures

### 

#### 

##### Plasmid Constructs

Plasmid constructs for the recombinant expression and purification of CaM (pMAL, New England Biolabs) or for overexpression of CaM in HEK293 cells (pcDNA3.1, Invitrogen) were prepared as described previously (8). Sanger sequencing verified the CaM-encoding inserts in all plasmids.

##### Endoplasmic Reticulum Luminal Ca^2+^ Imaging of HEK293 Cells Expressing RyR2

Stable expression of murine RyR2, or a RyR2 variant with the CaMBD deleted (murine ΔK3583-F3603), in HEK293 cells co-transfected with plasmids encoding CaM and the D1ER Ca^2+^ probe was done as described previously (27). Briefly, D1ER FRET signals reflecting ER luminal [Ca^2+^]_free_ in individual cells were monitored by using an epifluorescent microscope (27, 48). Each FRET signal trace was used to measure the Ca^2+^ release properties of the RyR2 channels relative to the ER Ca^2+^ store capacity: the activation and termination thresholds and their difference taken as the fractional Ca^2+^ release. The ER Ca^2+^ store capacities were calculated from the difference between maximum and minimum FRET signal (*F*_max_ − *F*_min_) (Fig. 2). The measured RyR2 Ca^2+^ release properties were compared using one-way ANOVA with Tukey's multiple comparisons test for all possible combinations, with adjusted *p* < 0.05 taken as significant. The experiments included a control without plasmid expression of CaM.

##### Estimation of CaM-WT and RyR2 Expression Levels in HEK293 Cells

HEK293 cells were cultured as described above with CaM-WT overexpression from a low (8) or high expressing plasmid (this study and Ref. 27) and without overexpression (Ctrl). Overexpression plasmids differed in their Kozak sequences upstream of the *CALM1* cDNA inserts. 40 μg of total protein from cell lysates (protein assay, Bio-Rad) was subjected to SDS-PAGE (1 h at 20 A) in a gradient gel (Bio-Rad, Mini-Protean TGX 4–20%) alongside a molecular weight marker (Bio-Rad, catalog No. 161-0309), and the electrophoretic separated proteins were blotted (0.5 h at 100 V, ∼0.33 A) to a nitrocellulose membrane in Tris-glycine buffer (Bio-Rad) with 10‰ SDS. The membrane was transiently stained with Ponceau S and cut into three regions: RyR2 (∼500 kDa), β-actin (∼42 kDa), and CaM (∼16 kDa). The membrane pieces were blocked in PBS with 1% casein (Bio-Rad), washed in PBS, and then incubated overnight with different primary antibodies (Ab) against CaM (05-173, EMD Milipore), β-actin (A5316, Sigma), or RyR2 (MA3-925, Pierce) in PBS with 1‰ Ab, 20% fetal bovine serum (Sigma), and 17 mm NaN_3_. After another wash, the pieces were incubated for 0.5 h with a secondary Ab (in-house anti-mouse IgG conjugated to horseradish peroxidase) and then washed again. The amount of bound secondary Ab was detected using luminol reagent (detection reagent 1–2, Thermo Scientific) with the resulting chemiluminescence imaged (ImageQuant LAS 4000, GE Healthcare Life Sciences). As a measure of protein expression levels, the protein band area intensities were quantified in ImageJ, and the expression levels of RyR2 and CaM in the individual samples were normalized to that of β-actin (49) (Fig. 8, *normalized area*). Western blotting analysis was done in at least duplicate.

##### Bilayer Recordings of Single RyR2 Channels

Native SR microsomes isolated from rat ventricular muscle were incorporated into bilayers using a modification of the method described by Chamberlain *et al.* (47, 50, 51). Briefly, planar lipid bilayers (50 mg/ml in a 5:4:1 mixture of bovine brain phosphatidylethanolamine, -serine, and -choline in *n*-decane) were formed across a 100-μm-diameter hole in a Teflon partition separating two compartments with cytosolic (114 mm Tris-HEPES, 5 mm ATP, 1 mm free Mg^2+^, 1 mm EGTA, and 10 μm free Ca^2+^ at pH 7.4) and luminal (cytosolic solution plus 200 mm Cs-HEPES and 1 mm free Ca^2+^) recording solutions. Single RyR2 activity was measured before and 20 min after the addition of CaM variants (1 μm) to the cytosolic solution. Recordings were made at room temperature (20–22 °C) with currents sampled at 50 μs/point and filtered at 0.75 kHz (4-pole Bessel). Analysis was done using pCLAMP9 software (Molecular Devices, Sunnyvale, CA). Recapitulating the cytosolic and intra-SR cellular milieu *in vitro* during planar lipid bilayer studies is impossible. Consequently, experimental compromises were necessary, and here the solutions approximated those in cardiomyocytes during systole. Low cytosolic Ca^2+^ (0.1–1 μm) reduces RyR2 activity to a level unsuitable for reliable measurements. Some researchers have overcome this issue by omitting Mg^2+^, but this causes a very non-physiological RyR2 Ca^2+^ dependence, as in cells Mg^2+^ normally competes with Ca^2+^ for occupancy of RyR2 cytosolic Ca^2+^ activation and inactivation sites (51). Differences in single channel parameters (*P_O_*, MOT, and MCT) extracted from time traces were compared using two-tailed *t* tests against the values following the addition of CaM-WT with *p* < 0.05 considered significant. Also, comparisons with control (no addition of CaM) were done with the same *p* value criteria.

##### Protein Expression and Purification

CaM was expressed from the pMAL vectors and purified as described previously (8). The identity, purity, and integrity of each protein preparation was confirmed by SDS-PAGE and MALDI-TOF mass spectrometry of trypsin-digested proteins.

##### CaM Ca^2+^ Titrations in the Presence of the RyR2(R3581-L3611) Peptide

A peptide corresponding to the RyR2 CaMBD (human RyR2 ^3581^RSKKAVWHKLLSKQRKRAVVACFRMAPLYNL^3611^) was purchased from Peptide 2.0 Inc. (Chantilly, VA) at > 98% purity. Titration experiments were done as described previously (8). Briefly; pH- and Ca ^2+^-buffered solutions (50 mm HEPES, 100 mm KCl, 0.5 mm EGTA, and 2 mm NTA at pH 7.2 (25 °C)) with or without 7 mm CaCl_2_ were mixed to obtain different [Ca ^2+^]_free_ levels (52). CaM (15 μm), RyR2(R3581-L3611) peptide (16.5 μm), 16.5 μm TCEP, and Fura-2 (Invitrogen) Ca^2+^ probe (0.8 μm) were added to double distilled water for dilution of 1.5× concentrated buffers. A 15% error for the [Ca^2+^]_free_ was included in data fitting procedures based on measuring [Ca^2+^]_free_ using Fura-2 and Ca^2+^ binding to CaM-WT. The intrinsic protein fluorescence from each CaM domain was monitored during CaM/RyR2 CaMBD complex Ca^2+^ titrations. The titration curves were fitted to a two-site Adair function as described previously (8, 19, 53, 54). Briefly, fluorescence intensities (FI) from the N- and C-domains of CaM were measured as partial Phe and Tyr emission spectra, and the fractional saturations (*Y*) for each domain were fitted to the raw FI according to Equation 1,

(Eq. 1) FI=Y×a+b

where *b* and *a* are the initial FI and the span in FI, respectively. *Y* is the fractional saturation of the monitored CaM domain binding two Ca^2+^ as described by the two-site Adair model,

(Eq. 2) $$Y=\frac{K_{1}\times \left[X\right]+2\times K_{2}\times {\left[X\right]}^{2}}{2\times \left(1+\times \left[X\right]+K_{2}\times {\left[X\right]}^{2}\right)}$$

where *K*_1_ is the sum of the microscopic equilibrium constants, and *K*_2_ is the equilibrium constant for the domain binding to two Ca^2+^. The apparent dissociation constant (app*K_D_*) for either domain was then calculated as the reciprocal square root of *K*_2_. The fitted *K*_2_ values were compared using one-way ANOVA with Dunnett's multiple comparison test against the value for CaM-WT/RyR2(R3581-L3611) titrations.

##### Isothermal Titration Calorimetry of RyR2(R3581-P3607) Peptide with CaM

A peptide corresponding to the CaMBD (human RyR2 ^3581^RSKKAVWHKLLSKQRKRAVVACFRMAP^3607^) was purchased from GenScript (Piscataway, NJ) at >95% purity. Titration of the RyR2(R3581-L3607) peptide with CaM was investigated under both Ca^2+^-free (apo) and Ca^2+^-saturating conditions and followed the use of ITC. Purified CaM variants were dialyzed (10 K Slide-a-lyzer^TM^, Thermo Scientific) against the titration buffer (10 mm HEPES, 150 mm KCl, pH 7.2) with either 10 mm EDTA or 10 mm CaCl_2_ added for 40 h at 4 °C (with buffer changed at 24 h). The spent dialysis buffer was filtered (0.22 μm) and used for diluting peptide and CaM preparations. 5 mm final TCEP was added to all solutions. Protein and peptide concentrations were estimated using absorption at 280 nm (NanoDrop ND-1000). Using an Auto-ITC200 (Malvern Instruments Ltd.) isothermal titration calorimeter, the peptide (apo 0.1 mm, Ca^2+^ 10 μm) in the sample cell was titrated 20 times at 25 °C with CaM (apo 1 mm, Ca^2+^ 100 μm) in 2-μl increments at 2-min intervals and with 750 rpm stirring (*c* values: apo 2–10 and Ca^2+^ 550–850). Two to three titrations were done for each combination of CaM variant and Ca^2+^ condition. ITC curves (change in instrument heat effect as a function of time, DP) were analyzed using MicroCal PEAQ-ITC software, and the extracted curves of the cumulative reaction heat as a function of total CaM concentration were fitted to a two-component binding equation (32). The reaction stoichiometry (*n*), standard enthalpy change (Δ*H*°), and dissociation constant (*K_D_*) were estimated from this fitting, and the parameters were compared using one-way ANOVA and Tukey's post hoc test for all possible comparisons. The binding reaction change in standard Gibb's free energy (Δ*G*°) and entropy contribution (−*T*·ΔS°) were calculated from Equation 3.

(Eq. 3) $$\Delta G^{O}+\Delta H^{O}-T\times \Delta S^{O}=-RT\times \text{In}\left(\frac{1}{K_{D}}\right)$$

##### NMR Spectra of ^15^N-Labeled RyR2(R3581-L3611) Peptide in Complex with CaM

A version of the RyR2 CaMBD peptide, ^15^N-labeled at Val-3586 and Phe-3603 (^15^N-RyR2(R3581-L3611)), was purchased from ProteoGenix (Schiltigheim, France) at >98% purity. The complex of this peptide with CaM-WT, -F142L, or -N98S was investigated under both Ca^2+^-free (*apo*) and Ca^2+^-saturating conditions using NMR. All four samples contained 20 mm HEPES, 100 mm KCl, 2 mm TCEP, 1 mm PMSF, 5% D_2_O, 40 μm TSP-d_4_, 100 μm ^15^N-RyR2(R3581-L3611), and 200 μm CaM-WT or -F142L along with either 10 mm CaCl_2_ or 10 mm EDTA. Protein and peptide concentrations were estimated using absorption at 280 nm (NanoDrop ND-1000) and quantitative NMR. The pH of the samples was adjusted to 6.50 ± 0.03 with either 1 m KOH or 1 m HCl, and the final sample volume was 550 μl. ^15^N-HSQC spectra were recorded on a 600-MHz Bruker AVIII at 298.1 K. TopSpin 3.2. was used for the acquisition and processing of the spectra. To assign the peaks in the ^15^N-HSQC, a 2D ^15^N-TOCSY-HSQC with a DIPSI2 mixing of 60 ms and a γB1/2π of 9.6 kHz was performed.

## Author Contributions

M. T. S., R. W., F. V. P., M. F., S. R. W. C., and M. T. O. designed the research. M. T. S., Y. L., K. T. L., A. N., X. T., C. H., and R. W. performed the experiments. M. T. S., R. W., K. T. L., C. H., M. F., and M. T. O. analyzed the data. M. T. S., M. F., S. R. W. C., and M. T. O. wrote the paper.

## Footnotes

2 The abbreviations used are:

CaM: calmodulin
CPVT: catecholaminergic polymorphic ventricular tachycardia
LQTS: long QT syndrome
RyR2: cardiac ryanodine receptor
SR: sarcoplasmic reticulum
CaMBD: CaM-binding domain
SOICR: store overload-induced Ca^2+^ release
GoF: gain of function
LoF: loss of function
ER: endoplasmic reticulum
Ctrl: control
MOT: mean open time
MCT: mean closed time
ITC: isothermal titration calorimetry
ANOVA: analysis of variance
TCEP: tris(2-carboxyethyl)phosphine
FI: fluorescence intensity
*P_O_*: open probability.
